# Supplementary material for: A longitudinal study of the association between attending cultural events and coronary heart disease
Source: Commun Med (Lond). 2023 May 24;3:72. doi: 10.1038/s43856-023-00301-0 (PMC10209104; doi:10.1038/s43856-023-00301-0)
Supplement: Supplementary file 1 — Description of Additional Supplementary Files [file 43856_2023_301_MOESM1_ESM.pdf]

Description of Additional Supplementary File

File Name: Supplementary Data

Description: Statistical codes used to obtain the results in the manuscript “A longitudinal study of the association between attending cultural events and coronary heart disease”
