# Supplementary material for: A longitudinal study of the association between attending cultural events and coronary heart disease
Source: Commun Med (Lond). 2023 May 24;3:72. doi: 10.1038/s43856-023-00301-0 (PMC10209104; doi:10.1038/s43856-023-00301-0)
Supplement: Supplementary file 2 — Supplementary Data [file 43856_2023_301_MOESM2_ESM.zip › 3. Supplementary Data_CODE_230507.docx]

**Supplementary Data**

*Legend:* Statistical codes used to obtain the results in the manuscript “A longitudinal study of the association between attending cultural events and coronary heart disease”

**Codes for results presented in Table 2.**

**Table 2.** Sex- and age-adjusted incidence rates per 1000 person-years of coronary heart disease (n=3296) and number of events (cases = 694) by the level of cultural exposure and the included confounders. STATA

**Note 1**

*poisson _d ib3.agek11 i.KON i.fkulindk,*

*margins fkulindk,predict(ir)*

*table fkulindk _d*

*poisson _d ib3.agek11 i.KON##ib2.fkulindk,*

*margins fkulindk,predict(ir)*

*poisson _d ib3.agek11 i.KON i.hregk,*

*margins hregk,predict(ir)*

*table hregk _d*

*poisson _d ib3.agek11 i.KON ib3.utbk,*

*margins utbk,predict(ir)*

*table utbk _d*

*poisson _d ib3.agek11 i.KON ib1.sambogiftk,*

*margins sambogiftk,predict(ir)*

*table sambogiftk _d*

*poisson _d ib3.agek11 i.KON ib0.smokek,*

*margins smokek,predict(ir)*

*table smokek _d*

*poisson _d ib3.agek11 i.KON i.all_motk,*

*margins all_motk,predict(ir)*

*table all_motk _d*

*poisson _d ib3.agek11 i.KON ib0.socisok,*

*margins socisok,predict(ir)*

*table socisok _d*

P-values within each level of the variables compared with a reference category in a sex-and age-adjusted Poisson model, **note 2** in the table.

*stcox ib2.KON ib3.agek11 if inkl==1,vce(cluster LOPNR)*

*stcox ib2.KON ib3.agek11 ib3.utbk if inkl==1,vce(cluster LOPNR)*

*stcox ib2.KON ib3.agek11 ib1.sambogiftk if inkl==1,vce(cluster LOPNR)*

*stcox ib2.KON ib3.agek11 ib0.socisok if inkl==1,vce(cluster LOPNR)*

*stcox ib2.KON ib3.agek11 i.hregk if inkl==1,vce(cluster LOPNR)*

*stcox ib2.KON ib3.agek11 i.smokek if inkl==1,vce(cluster LOPNR)*

*stcox ib2.KON ib3.agek11 i.all_motk if inkl==1,vce(cluster LOPNR)*

*stcox ib2.KON ib3.agek11 ib0.fkulindk if inkl==1,vce(cluster LOPNR)*

**Codes for results presented in Table 3.**

**Table 3.** The association between cultural exposure and coronary heart disease in a longitudinal study of three separate measurements with eight-year intervals (n = 3296)

Cox proportional hazard regression, full model, see **notes 1 and 2**, STATA

*stcox ib2.KON#ib3.agek11 ib0.fkulindk i.hregk ib1.sambogiftk ib0.socisok ib0.smokek ib3.utbk i.all_motk if inkl==1,vce(cluster LOPNR)*

*estat phtest, detail* /* test of proportionality*/

MSM-Cox with IPW, see **notes 1 and 3,** R**.**

First, we estimated inverse probability weights (IPW) based on the same confounders as included in stcox above.

Dataset<- load("D:/usb/Kultur_Olle_Bygren/CHD/Kultur_split_2021122_CHD_R_R.RData")

*library("ipw")*

*temp <- ipwtm(exposure = fkulindk,*

*family = "binomial", link = "logit",*

*numerator = ~ fkulindk_lag + KON,*

*denominator = ~ ns(alder1, df=4) +*

*fkulindk_lag + factor(hregk_lag) + sambogiftk_lag + socisok_lag + smokek_lag + factor(utbk_lag) + all_motk_lag,*

*id = LOPNR, timevar = X_t, type = "all",*

*data = Dataset)*

Thereafter we run Cox regression within R,

*library (“survival”)*

*summary(coxph(Surv(X_t0, X_t, X_d) ~ factor(fkulindk) + cluster(LOPNR),*

*data = Dataset, weights= sw3))*

*exp(coef) exp(-coef) lower .95 upper .95*

*factor(fkulindk)[T.1] 0.8002 1.250 0.6552 0.9773*

*factor(fkulindk)[T.2] 0.6602 1.515 0.5046 0.8639*

*summary(Dataset$sw3)*

*Min. 1st Qu. Median Mean 3rd Qu. Max.*

*0.3277 0.7311 0.9222 1.0002 1.0569 14.4230*

Principal factor analysis was run three times, one for each measurement to estimate the cultural participation index,

*82/83=0; STATA was used

*polychoric BIO0 TEATER0 KONSERT0 KONSTHALL0 MUSEUM0 GUDSTJANST0*

*scalar fa_N = r(N)*

*matrix fa_r = r(R)*

*collapse (mean) BIO0 TEATER0 KONSERT0 KONSTHALL0 MUSEUM0 GUDSTJANST0*

*mkmat BIO0 TEATER0 KONSERT0 KONSTHALL0 MUSEUM0 GUDSTJANST0, matrix(fa_m)*

*use "D:\usb\Kultur_Olle_Bygren\CHD\Kultur_WIDE1_20211122_CHD.dta",clear*

*collapse (sd) BIO0 TEATER0 KONSERT0 KONSTHALL0 MUSEUM0 GUDSTJANST0*

*mkmat BIO0 TEATER0 KONSERT0 KONSTHALL0 MUSEUM0 GUDSTJANST0, matrix(fa_s)*

*use "D:\usb\Kultur_Olle_Bygren\CHD\Kultur_WIDE1_20211122_CHD.dta",clear*

*pcamat fa_r, n(`=fa_N') means(fa_m) sds(fa_s) factors(1)*

*rotate*

*drop pca11_0*

*predict pca11_0*

*su pca11_0*

**su pca12*

*drop perc_pca11_0*

*egen perc_pca11_0 = cut(pca11_0), group(4)*

*tab perc_pca11_0*

*replace perc_pca11_0=1 if perc_pca11_0==2*

*replace perc_pca11_0=2 if perc_pca11_0==3*

*save "D:\usb\Kultur_Olle_Bygren\CHD\Kultur_WIDE1_20211122_CHD.dta",replace*

*90/91=8; STATA was used

*polychoric BIO8 TEATER8 KONSERT8 KONSTHALL8 MUSEUM8 GUDSTJANST8*

*scalar fa_N = r(N)*

*matrix fa_r = r(R)*

*collapse (mean) BIO8 TEATER8 KONSERT8 KONSTHALL8 MUSEUM8 GUDSTJANST8*

*mkmat BIO8 TEATER8 KONSERT8 KONSTHALL8 MUSEUM8 GUDSTJANST8, matrix(fa_m)*

*use "D:\usb\Kultur_Olle_Bygren\CHD\Kultur_WIDE1_20211122_CHD.dta",clear*

*collapse (sd) BIO8 TEATER8 KONSERT8 KONSTHALL8 MUSEUM8 GUDSTJANST8*

*mkmat BIO8 TEATER8 KONSERT8 KONSTHALL8 MUSEUM8 GUDSTJANST8, matrix(fa_s)*

*use "D:\usb\Kultur_Olle_Bygren\CHD\Kultur_WIDE1_20211122_CHD.dta",clear*

*pcamat fa_r, n(`=fa_N') means(fa_m) sds(fa_s) factors(1)*

*rotate*

*drop pca11_8*

*predict pca11_8*

*su pca11_8*

**su pca12*

*drop perc_pca11_8*

*egen perc_pca11_8 = cut(pca11_8), group(4)*

*tab perc_pca11_8*

*replace perc_pca11_8=1 if perc_pca11_8==2*

*replace perc_pca11_8=2 if perc_pca11_8==3*

*save "D:\usb\Kultur_Olle_Bygren\CHD\Kultur_WIDE1_20211122_CHD.dta",replace*

*98/99=16; STATA was used

*polychoric BIO16 TEATER16 KONSERT16 KONSTHALL16 MUSEUM16 GUDSTJANST16*

*scalar fa_N = r(N)*

*matrix fa_r = r(R)*

*collapse (mean) BIO16 TEATER16 KONSERT16 KONSTHALL16 MUSEUM16 GUDSTJANST16*

*mkmat BIO16 TEATER16 KONSERT16 KONSTHALL16 MUSEUM16 GUDSTJANST16, matrix(fa_m)*

*use "D:\usb\Kultur_Olle_Bygren\CHD\Kultur_WIDE1_20211122_CHD.dta",clear*

*collapse (sd) BIO16 TEATER16 KONSERT16 KONSTHALL16 MUSEUM16 GUDSTJANST16*

*mkmat BIO16 TEATER16 KONSERT16 KONSTHALL16 MUSEUM16 GUDSTJANST16, matrix(fa_s)*

*use "D:\usb\Kultur_Olle_Bygren\CHD\Kultur_WIDE1_20211122_CHD.dta",clear*

*pcamat fa_r, n(`=fa_N') means(fa_m) sds(fa_s) factors(1)*

*rotate*

*drop pca11_16*

*predict pca11_16*

*su pca11_16*

*drop perc_pca11_16*

*egen perc_pca11_16 = cut(pca11_16), group(4)*

*tab perc_pca11_16*

*replace perc_pca11_16=1 if perc_pca11_16==2*

*replace perc_pca11_16=2 if perc_pca11_16==3*

*save "D:\usb\Kultur_Olle_Bygren\CHD\Kultur_WIDE1_20211122_CHD.dta",replace*
