# Supplementary material for: A longitudinal study of the association between attending cultural events and coronary heart disease
Source: Commun Med (Lond). 2023 May 24;3:72. doi: 10.1038/s43856-023-00301-0 (PMC10209104; doi:10.1038/s43856-023-00301-0)
Supplement: Supplementary file 4 — Reporting Summary [file 43856_2023_301_MOESM4_ESM.pdf]

nature research

Corresponding author(s): Filip Jansåker
Last updated by author(s): Apr 4, 2023

Reporting Summary

Nature Research wishes to improve the reproducibility of the work that we publish. This form provides structure for consistency and transparency in reporting. For further information on Nature Research policies, see our Editorial Policies and the Editorial Policy Checklist.

Statistics

For all statistical analyses, confirm that the following items are present in the figure legend, table legend, main text, or Methods section.

n/a	Confirmed	
☐	☒	The exact sample size (*n*) for each experimental group/condition, given as a discrete number and unit of measurement
☐	☒	A statement on whether measurements were taken from distinct samples or whether the same sample was measured repeatedly
☐	☒	The statistical test(s) used AND whether they are one- or two-sided<br>*Only common tests should be described solely by name; describe more complex techniques in the Methods section.*
☐	☒	A description of all covariates tested
☐	☒	A description of any assumptions or corrections, such as tests of normality and adjustment for multiple comparisons
☐	☒	A full description of the statistical parameters including central tendency (e.g. means) or other basic estimates (e.g. regression coefficient) AND variation (e.g. standard deviation) or associated estimates of uncertainty (e.g. confidence intervals)
☐	☒	For null hypothesis testing, the test statistic (e.g. *F*, *t*, *r*) with confidence intervals, effect sizes, degrees of freedom and *P* value noted<br>*Give P values as exact values whenever suitable.*
☒	☐	For Bayesian analysis, information on the choice of priors and Markov chain Monte Carlo settings
☐	☒	For hierarchical and complex designs, identification of the appropriate level for tests and full reporting of outcomes
☐	☒	Estimates of effect sizes (e.g. Cohen's *d*, Pearson's *r*), indicating how they were calculated

Our web collection on statistics for biologists contains articles on many of the points above.

Software and code

Policy information about availability of computer code

Data collection	STATA. StataCrop.Stata Statistical Sfotware: Release 16. SataCorp LLC, 2019.
Data analysis	STATA. StataCrop.Stata Statistical Sfotware: Release 16. SataCorp LLC, 2019. & R. R Core Team. R. Www.r-project.org 2013.<br>van der Wal et al. IPW: An R Package for Inverse Probability Weighting. Journal of Satistical Software 43. 2011.

For manuscripts utilizing custom algorithms or software that are central to the research but not yet described in published literature, software must be made available to editors and reviewers. We strongly encourage code deposition in a community repository (e.g. GitHub). See the Nature Research guidelines for submitting code & software for further information.

Data

Policy information about availability of data

All manuscripts must include a data availability statement. This statement should provide the following information, where applicable:
- Accession codes, unique identifiers, or web links for publicly available datasets
- A list of figures that have associated raw data
- A description of any restrictions on data availability

A description of restrictions on data availability is included.

Field-specific reporting

Please select the one below that is the best fit for your research. If you are not sure, read the appropriate sections before making your selection.

☒ Life sciences ☐ Behavioural & social sciences ☐ Ecological, evolutionary & environmental sciences

For a reference copy of the document with all sections, see nature.com/documents/nr-reporting-summary-flat.pdf

Life sciences study design

All studies must disclose on these points even when the disclosure is negative.

Sample size	This study aimed to examine the longitudinal effect between cultural participation and coronary heart disease (CHD) in a randomly selected representative adult cohort (n=3296) of the Swedish population. The study period was 36 years (1982–2017) with three separate eight-year interval measurements of cultural exposure (e.g. visiting theaters and museums) starting in 1982/83. The study included participants with complete data from all three measurement points between 1982-2017 and no CHD prior to the study.
Data exclusions	Participants with the outcome (CHD) prior to the start of the study period were excluded (N=15), resulting in 3296 participants in the study population for the analysis.
Replication	Original study
Randomization	Observational study.
Blinding	Observational study.

Reporting for specific materials, systems and methods

We require information from authors about some types of materials, experimental systems and methods used in many studies. Here, indicate whether each material, system or method listed is relevant to your study. If you are not sure if a list item applies to your research, read the appropriate section before selecting a response.

Materials & experimental systems

n/a	Involved in the study	
☒	☐	Antibodies
☒	☐	Eukaryotic cell lines
☒	☐	Palaeontology and archaeology
☒	☐	Animals and other organisms
☐	☒	Human research participants
☒	☐	Clinical data
☒	☐	Dual use research of concern

Methods

n/a	Involved in the study	
☒	☐	ChIP-seq
☒	☐	Flow cytometry
☒	☐	MRI-based neuroimaging

Human research participants

Policy information about studies involving human research participants

Population characteristics	See table 1 in manuscript.
Recruitment	See methodology. In short: The study population was collected from the Statistics on Income and Living Conditions. Only participants with three measurements and without coronary heart disease (CHD) occurring before the start of the study (n=15) were included in the analysis (n=3296). Data were split into one-year intervals and originated from the following years: 1982/83, 1990/91, and 1998/99; the follow-up ended on the 31st of December 2017.
Ethics oversight	This study was a non-intervention register study on already collected and encrypted secondary data. It was conducted according to the guidelines of the Declaration of Helsinki and approved by the Ethical Review Board in Lund and was exempted from informed consent requirements owing to its register based design. Access to the used registries was obtained from Swedish authorities prior to the study commencing and all methods were used in accordance with national guidelines and regulations.

Note that full information on the approval of the study protocol must also be provided in the manuscript.
